# Iron Deficiency and Anemia 10 Years After Roux-en-Y Gastric Bypass for Severe Obesity

**DOI:** 10.3389/fendo.2021.679066

**Published:** 2021-09-22

**Authors:** Jorunn Sandvik, Kirsti Kverndokk Bjerkan, Hallvard Græslie, Dag Arne Lihaug Hoff, Gjermund Johnsen, Christian Klöckner, Ronald Mårvik, Siren Nymo, Åsne Ask Hyldmo, Bård Eirik Kulseng

**Affiliations:** ^1^Department of Surgery, Møre and Romsdal Hospital trust, Ålesund, Norway; ^2^Centre for Obesity Research, Clinic of Surgery, St. Olav’s University Hospital, Trondheim, Norway; ^3^Department of Clinical and Molecular Medicine, Faculty of Medicine and Health Sciences, Norwegian University of Science and Technology, Trondheim, Norway; ^4^Faculty of Social Science and History, Volda University College, Volda, Norway; ^5^Clinic of Surgery, Namsos Hospital, Nord-Trøndelag Hospital Trust, Namsos, Norway; ^6^Department of Research and Innovation, Møre and Romsdal Hospital Trust, Ålesund, Norway; ^7^Norwegian National Advisory Unit on Advanced Laparoscopic Surgery, Clinic of Surgery, St. Olav’s University Hospital, Trondheim, Norway; ^8^Department of Psychology, Norwegian University of Science and Technology, Trondheim, Norway

**Keywords:** iron deficiency, RYGB bypass, bariatric surgery, iron deficiency and anemia after RYGB, long-term RYGB, fatigue, intravenous iron, post-bariatric iron

## Abstract

Iron deficiency with or without anemia is a well-known long-term complication after Roux-en-Y, gastric bypass (RYGB) as the procedure alters the gastrointestinal absorption of iron. Iron is essential for hemoglobin synthesis and a number of cellular processes in muscles, neurons, and other organs. Ferritin is the best marker of iron status, and in a patient without inflammation, iron deficiency occurs when ferritin levels are below 15 µg/L, while iron insufficiency occurs when ferritin levels are below 50 µg/L. Lifelong regular blood tests are recommended after RYGB, but the clinical relevance of iron deficiency and iron insufficiency might be misjudged as long as the hemoglobin levels are normal. The aim of this study was to explore the frequency of iron deficiency and iron deficiency anemia one decade or more after RYGB, the use of per oral iron supplements, and the frequency of intravenous iron treatment. Nine hundred and thirty patients who underwent RYGB for severe obesity at three public hospitals in Norway in the period 2003–2009 were invited to a follow-up visit 10–15 years later. Results from blood tests and survey data on the use of oral iron supplements and intravenous iron treatment were analyzed. Ferritin and hemoglobin levels more than 10 years after RYGB were available on 530 patients [423 (79.8%) women]. Median (IQR) ferritin was 33 (16–63) µg/L, and mean (SD) hemoglobin was 13.4 (1.3) g/dl. Iron deficiency (ferritin ≤ 15 µg/L) was seen in 125 (23.6%) patients; in addition, iron insufficiency (ferritin 16–50 µg/L) occurred in 233 (44%) patients. Mean (SD) hemoglobin levels were 12.5 (1.4) g/dl in patients with iron deficiency, 13.5 (1.2) g/dl in patients with iron insufficiency, 13.8 (1.3) g/dl in the 111 (21%) patients with ferritin 51–100 µg/L, and 13.8 (1.2) g/dl in the 55 (10%) patients with ferritin >100 µg/L. Two hundred and seventy-five (56%) patients reported taking oral iron supplements, and 138 (27.5%) had received intravenous iron treatment after the RYGB procedure. Iron deficiency or iron insufficiency occurred in two-thirds of the patients 10 years after RYGB, although more than half of them reported taking oral iron supplements.

## Introduction

Anemia caused by iron deficiency is a commonly reported long-term complication from Roux-en-Y gastric bypass (RYGB) and other bariatric surgery methods, as these procedures alter the gastrointestinal absorption of iron and other minerals (1–3). However, anemia first occurs at a late stage in the process of decreasing iron stores, and iron deficiency without anemia might be an important cause of impaired health in patients who have undergone bariatric surgery (4). The clinical significance of iron deficiency without anemia is poorly understood, as the symptoms develop over time and might be caused by other physical or mental conditions (5, 6).

Iron is essential in hemoglobin synthesis, and in a number of cellular processes in muscles, neurons, and other organs (7). Optimal mitochondrial activity and energy production depend on available iron (8). Excess iron is toxic to the human body, and iron absorption, storage, and recycling of iron are therefore strictly regulated (9). Iron is mainly absorbed in the duodenum and proximal jejunum, parts of the gastrointestinal tract that are bypassed after RYGB. The hepatic hormone hepcidin plays a key role in regulating the uptake of iron from the intestinal lumen to the enterocytes, and in the transport of iron from the enterocytes to the blood (10, 11). Hepcidin is suppressed under iron deficiency, supporting excess iron absorption from the gut, if available. Biological processes, such as inflammation, that stimulate hepcidin expression, will reduce iron absorption even in a state of iron deficiency (12, 13).

Symptoms of iron deficiency without anemia include fatigue, reduced exercise performance, restless legs, and cognitive impairment (14–16). These diffuse symptoms are often registered as a late consequence of bariatric surgery, but the relationship between iron deficiency and these symptoms is not sufficiently understood (4, 6, 17).

In clinical settings, the iron transport molecule ferritin has proved to be the most reliable marker of iron status in patients without inflammation (18, 19). According to the World Health Organization (WHO), iron stores are considered depleted when ferritin levels are below 15 µg/L in adults (20). This cutoff is often used in clinical settings as well as in research to define iron deficiency, even if gender-specific and a higher cutoffs might be more appropriate to define the condition (21).

Low ferritin levels only occur under iron deficiency; however, ferritin is also an acute phase substance, meaning that iron deficiency can occur even if ferritin levels are normal or high (22, 23).

The definition of anemia differs between different populations. At present, hemoglobin levels of 13.7 g/dl in men and 12.0 g/dl in women are regarded as thresholds for anemia at the hospitals contributing to the present study. Iron status is one of several factors influencing hemoglobin levels; other factors might be inflammation, medication, or endocrine conditions (24–26).

So far, no ideal program for iron supplementation following bariatric surgery is agreed upon (27). International guidelines recommend per oral iron supplementation either as continuous or intermittent treatment for all patients undergoing RYGB to avoid iron deficiency (28–30). Annual blood tests are recommended lifelong after RYGB, but the clinical relevance of iron deficiency (ferritin levels < 15 µg/L) and iron insufficiency (ferritin levels 15–50 µg/L) might be misjudged as long as hemoglobin levels are normal.

Intravenous iron treatment is an alternative when per oral treatment is inadequate or not appropriate (31). As many as 25% of RYGB patients may need intravenous iron treatment to prevent anemia in the first 5 years after surgery (2, 32, 33).

The Bariatric surgery observation study (BAROBS) was conducted at three hospitals in Central Norway Health region in 2018–2020, in order to assess the general health status a decade after RYGB as treatment for severe obesity. The participants underwent a clinical examination and answered a survey on health and social issues. Included in the survey were questions on diffuse symptoms like muscular pain, fatigue, dizziness, restless legs, headache, and memory problems, as well as questions on compliance to vitamin and mineral supplement recommendations.

The main aim of the present study was to use data from BAROBS to explore the frequency of iron deficiency and iron deficiency anemia one decade or more after RYGB in a population with standardized follow-up in a public hospital setting the first 5 years after surgery, including education on the importance of lifelong vitamin and mineral supplements. Secondary aims were to explore the present use of per oral iron supplements, the frequency of intravenous iron treatment the following 10 to 15 years after RYGB surgery, as well as the relationship between iron deficiency and hemoglobin levels a decade after RYGB. The relationship between iron deficiency and self-reported symptoms, like fatigue, headache, restless legs and memory problems was also explored, and to the extent one could be found.

## Materials and Methods

This is a cross-sectional observational study of a bariatric surgery population supplemented by retrospective analysis of prospectively collected data on patients from three hospitals in Central Norway who underwent Roux-en-Y gastric bypass as treatment for severe obesity from 2003 to the end of 2009.

The criteria for being offered RYGB were for patients to be an adult (18–60 years), with BMI > 40 kg/m^2^ or alternatively BMI > 35 kg/m^2^ with obesity-related comorbidities, according to national and international guidelines at the time (34).

The study was conducted according to the guidelines laid down in the Declaration of Helsinki. All participants provided written informed consent before commencement. The study was approved by the Regional Ethics committee (REK 2017/1828 REK south-east B).

### Surgical Method and Follow-Up Program

The RYGB procedure was performed laparoscopically, and the antecolic Roux-limb was 100 cm in patients with BMI < 50 kg/m^2^, and 150 cm in patients with BMI > 50 kg/m^2^ prior to surgery. All patients were offered a standardized follow-up program at the operating hospital for up to 5 years, and lifelong vitamin and mineral supplements were recommended. The over-the-counter multivitamin and mineral-supplements, which were recommended to the patients on a daily basis after gastric bypass surgery, contains 15 mg iron (II) fumarate. Additional iron supplement was recommended on an individual basis as continuous or intermittent treatment to keep the ferritin levels above 50 µg/L, and the patients were advised to use whichever over-the-counter iron supplement they preferred. When intravenous iron treatment was needed, it was mainly given as a single dose of 1 g of ferric carboxymaltose, or less often as iron sucrose 200 mg over five visits. During the postoperative observation period, several oral iron supplements have been available without prescription from a physician, and participants reported only current supplement practice.

As part of the BAROBS study, a set of blood tests, including hemoglobin and iron status, were taken. A standardized set of blood tests had been taken before the RYGB procedure, and at 6, 12, 18, 24, 36, 42, and 60 months after the surgery. The same set of blood tests were taken, if clinically relevant, the following years until they were included in the BAROBS study. The participants consented to extraction of historical data from the laboratory files at the three hospitals, including all blood test results related to the obesity treatment pre- and postoperatively. Annual follow-up with blood tests by a general practitioner was recommended after five years. These results were not included in this study, unless a copy of the blood test results was sent to the local obesity clinic.

### Biochemical Assessments

Fasting venous blood was analyzed just like other routine blood samples by standard accredited methods at the Departments of Medical Biochemistry at St. Olav’s University Hospital, Trondheim, Ålesund Hospital, and Namsos Hospital.

Ferritin levels were analyzed by an immunochemical assay delivered by Roche Cobas (Rotkreuz, Switzerland) or Abbott Alinity (Illinois, USA).

Hemoglobin levels were analyzed by a photometric method from Sysmex XN (Kobe, Japan).

C-reactive protein (CRP) levels were analyzed by an immunological assay from Siemens Advia Chemistry XP, or Cobas c702 and c501 (Roche Diagnostics, Rotkreuz, Switzerland), or an immune turbid metric assay from Abbott Alinity (Illinois, USA).

Testosterone levels were analyzed by high-pressure liquid chromatography (HPLC) by the Agilent 1290/Agilent 6465 Triple Quad LC/MS-MS (Santa Clara, USA), or electro-chemiluminescence assay (ECLIA) using Cobas e801 (Roche Diagnostics, Rotkreuz, Switzerland).

### Statistical Methods

Continuous normal distributed variables are reported as mean ± standard deviation (SD), and for these variables, independent *t*-tests have been performed. The non-normal distributed variables are reported as median with interquartile range (IQR), and for these variables, non-parametric Mann–Whitney *U* tests have been performed. Categorical variables are reported with numbers and percentages, and for these variables, Chi-squared tests have been performed. Bivariate correlation analyses were performed to explore the relationships between continuous variables. Bonferroni correction for multiple hypothesis testing has been applied and reported as needed. Differences were considered significant at *p* < 0.05.

The statistical analysis has been performed using IBM SPSS version 27 (SPSS Inc., Chicago, IL, USA).

## Results

Out of a total of 959 patients who underwent RYGB surgery during the period, 29 patients died during the observation period. The remaining 930 patients were eligible for inclusion, and they were invited to a follow-up visit between August 2018 and June 2020. Five hundred and forty-six patients (58.7%) appeared for their follow-up visit and were included in the BAROBS study. Fifteen patients who had RYGB as a secondary bariatric procedure and one patient who did not take the blood tests were excluded from the analyses presented in this paper. Four hundred and twenty-three (79.8%) of the remaining 530 participants were women.

There was no difference in age at RYGB surgery between BAROBS participants, with a mean age at 40.1 years, and the eligible population, with a mean age at 39.6 years. The participation rate was somewhat higher among women than men, as there were 212/959 (22.1%) men in the population, as compared to 107/530 (20.2%) among the BAROBS participants (n.s.).

For women, the mean age at RYGB surgery was 39.9 (8.9) years and 41.1 (9.4) years for men. Furthermore, the mean age at follow-up visit was 51.6 (9.0) years for women and 52.8 (9.1) years for men. The mean preoperative BMI was 44.2 (5.2) kg/m^2^ for women and 45.1(6.3) kg/m^2^ for men, and the mean observational time was 141 (20) months (**Table 1**).

**Table 1:** Patients’ characteristics.

	Women (423)	Men (107)	*p*
Age at baseline, years Mean (SD)	39.9 (8.9)	41.1 (9.4)	0.228
Age at follow-up, years Mean (SD)	51.6 (9.0)	52.8 (9.1)	0.227
Preoperative BMI, kg/m^2^ Mean (SD)	44.2 (5.2)	45.1 (6.3)	0.168
%TWL at nadir Mean (SD)	33.2 (8.6)	32.9 (10.7)	0.787
Hemoglobin, g/dl* Mean (SD)	13.1 (1.2)	14.5 (1.3)	****
Ferritin, µg/L** Median (IQR)	33 (16-63)	41 (20-81)	****
Testosterone, nmol/L Mean(SD)***	0.79 (0.41)	16.5 (6.5)	****

*Local reference values hemoglobin: women 12.0–15.3 g/dl, men 13.7–17.0 g/dl.

**Local reference values ferritin: women 20–167 µg/L, men 30–383 µg/L.

***Local reference values testosterone: women 0.34–1.40 nmol/L, men 6.73–31.88 nmol/L.

**** Since the reference values are different between men and women for these measurements, no comparison has been made.

Median (IQR) ferritin level was 33 (16–63) µg/L for all participants, 41 (20–81) µg/L in men, and 30 (15–57) µg/L in women. Iron deficiency (ferritin ≤ 15 µg/L) was seen in 125 (23.6%) of all participants, as this condition occurred in 106/423 (25.1%) women and 19/107 (17.8%) men (n.s.). In addition, iron insufficiency (ferritin 16–50 µg/L) occurred in 233 (44%) of all patients, 44/107 (41.1%) men, and 189/423 (44.7%) women (n.s.) (**Table 2**).

**Table 2:** Frequency of iron deficiency, anemia, iron supplements, and related symptoms in men and women 10–15 years after RYGB-surgery.

	Men	Women	*p*
Total number of patients *N* = 530	*N* = 107	*N* = 423	
Ferritin < 15 µg/L, *N* (%) *N* = 530 (107/423)	19 (17.8)	106 (25.1)	0.112
Ferritin < 50 µg/L, *N* (%) *N* = 530 (107/423)	63 (58.9)	295 (69.7)	0.032**
Anemia *N* (%) *N* = 530 (107/423)	26 (24.3)	68/423 (16.1)	0.046**
Per oral iron *N* (%) *N = 494 (102/392)	47 (46)	228 (58.2)	0.029**
Intravenous iron *N* (%) *N = 501 (102/398)	14(13.6)	124(31.2)	<0.001
Fatigue *N* (%) *N = 495 (102/393)	37 (36.3)	238 (60.6)	<0.001
Restless legs *N* (%) *N = 500 (104/396)	37 (35.6)	189 (47.7)	0.027**
Muscular pain *N* (%) *N = 497 (101/396)	50 (50.5)	281 (71.0)	<0.001
Dizziness *N* (%) *N = 497 (102/395)	12 (11.8)	151(38.2)	<0.001
Headache *N* (%) *N = 501 (103/398)	37 (35.9)	224 (56.3)	<0.001

*N refers to the actual number of patients responding to these questions in the survey.

**These p-values do not reach the level of significance when correcting for multiple hypothesis testing.

The mean (SD) hemoglobin level was 13.4 (1.3) g/dl in the entire population, 13.1 (1.2) g/dl in women, and 14.5 (1.3) g/dl in men. Regarding the frequency of anemia, according to the local gender-specific definitions, 68 (16.1%) women had hemoglobin below 12.0 g/dl and 26 (24.3%) men had hemoglobin below 13.7 g/dl (*p* = 0.047), which is not a significant difference when correcting for multiple hypothesis testing. Mean (SD) hemoglobin levels were 12.5 (1.4) g/dl in the 125 (23.6%) patients with iron deficiency, 13.5 (1.2) g/dl in the 233 (44.0%) patients with iron insufficiency, 13.8 (1.3) g/dl in the 111 (21.0%) patients with ferritin 51–100 µg/L, and 13.8 (1.2) g/dl in the 61 (11.5%) patients with ferritin >100 µg/L.

**Figure 1:**
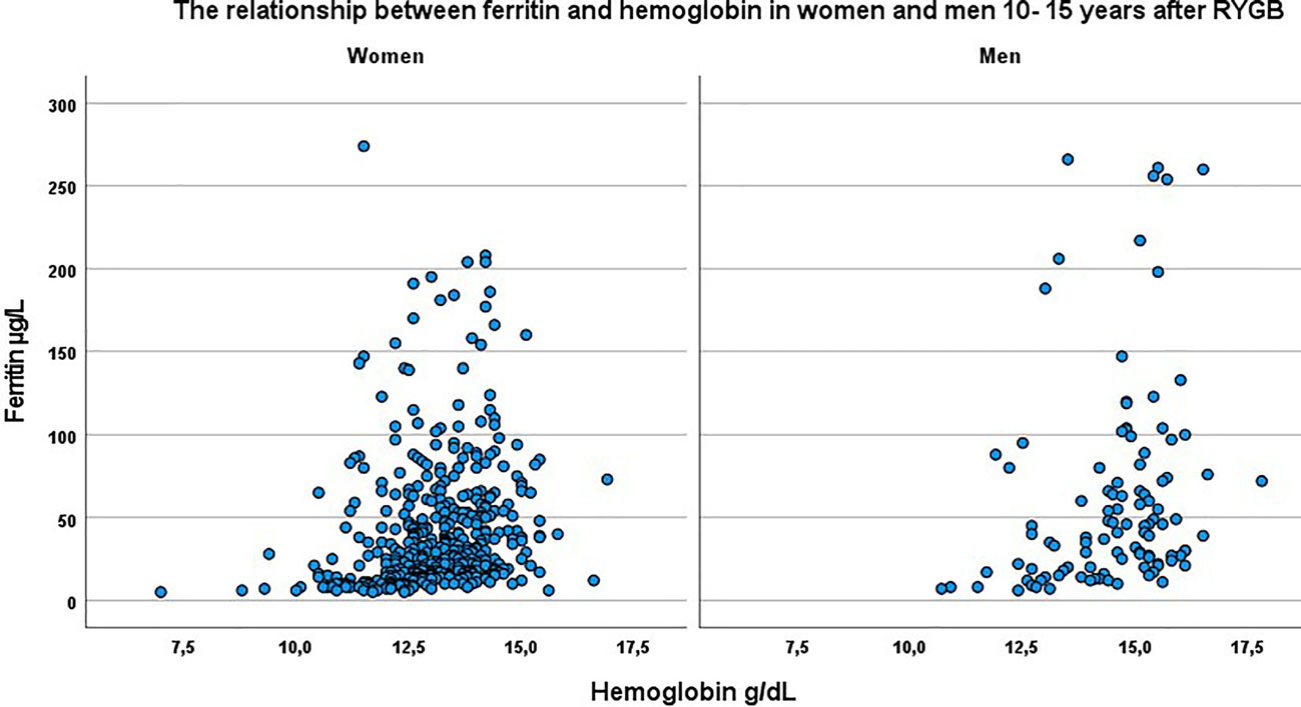
The relationship between ferritin and hemoglobin in women and men 10-15 years after RYGB.

When comparing the hemoglobin levels in women and men, with and without iron deficiency, hemoglobin in women was at 12.4 (1.4) g/dl when ferritin was at ≤ 15 µg/L, and at 13.3 (1.0) g/dl when ferritin was at > 15 µg/L (*p* < 0.001). In men, hemoglobin was at 13.3 (1.3) g/dl when ferritin was at ≤ 15 µg/L, and at 14.8 (1.2) g/dl when ferritin was at > 15 µg/L (*p* < 0.001). The relationship between ferritin and hemoglobin among men and women is visualized in **Figure 1**, illustrating that hemoglobin levels are normal for a high proportion of participants with low iron levels.

Self-reported information on use of iron supplementation at present was available for 494 patients, and 275 (56%) patients reported taking per oral iron supplements on a regular basis. One hundred and six (21.4%) took iron supplements daily or several times a week, and 170 (34.3%) took them periodically, e.g., 1 month twice or more a year, at the time of follow-up. Overall, median ferritin was 34 (16-64) µg/L in patients taking per oral iron supplements and 29 (16-58) µg/L in patients who did not (n.s.).

Women (228/392) on iron supplements had hemoglobin levels of 13.0 (1.0) g/dl, compared to hemoglobin levels of 13.2 (1.1) g/dl in women without iron supplements (n.s.). Hemoglobin was at 14.4 (1.3) g/dl in men (47/102) on iron supplements, and at 14.7(1.3) g/dl in men without (n.s.).

Information on intravenous iron treatment in the years following the RYGB procedure was available for 501 participants, and 138 (27.5%) had received this treatment. This was more common among women 124/398 (31%) than men 14/103 (14%) (*p* < 0.001). Median (IQR) ferritin was 38 (16–66) µg/L in patients who had received intravenous iron treatment once or several times, and 30 (16–60) µg/L in patients who had not (n.s.). Hemoglobin was 13.1 (1.1) g/dl in women and 14.1 (1.4) g/dl in men who had received intravenous iron treatment, compared to 13.1 (1.2) g/dl in women and 14.6 (1.3) g/dl in men without.

**Figure 2:**
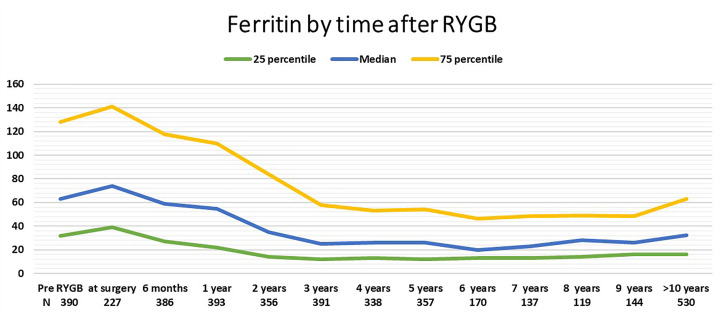
Median and interquartile range of ferritin by time after RYGB.

In the historical data on blood test results related to obesity treatment pre- and postoperatively, ferritin levels measured at different points from before the RYGB surgery and up to 5 years after were available for 227–391 (43%–74%) patients. In addition, ferritin levels after 6 to 9 years were available for 119–170 (22%–32%) patients. There was no difference in gender, age, or BMI at surgery, among patients with and without ferritin results at the specific times, and we therefore decided to include the results for time points with less available data for illustrative purposes. Median (IQR) levels of ferritin from before RYGB up to the last follow-up are presented in **Figure 2**. Missing data might be due to blood tests analyzed outside the hospitals, technical problems when extracting laboratory data from more than 10 years ago, or patients not meeting to the planned follow-up.

When exploring the relationship between two different threshold values of ferritin and symptoms like fatigue, headache, dizziness, and restless legs, no associations between these symptoms at ferritin above or beyond 15 µg/L or 50 µg/L were found (**Table 3**).

**Table 3:** Iron supplements and symptoms possibly related to low iron stores one decade after RYGB, at two different threshold values of ferritin.

*N* = 530	Ferritin < 15 *N* = 125	Ferritin > 15 *N* = 405	*p*	Ferritin < 50 *N* = 358	Ferritin > 50 *N* = 172	*p*
Per oral iron *N = 494, *N* (%)	66/119 (55.4)	209/375 (55.7)	0.959	181/334 (54.2)	94/160 (59.6)	0.340
Intravenous iron *N = 501, *N* (%)	33/122 (27.0)	105/379 (27.7)	0.888	85/340 (25.0)	53/161 (32.9)	0.064
Fatigue *N = 495, *N* (%)	63/119 (52.9)	212/376 (56.4)	0.510	176/336 (52.4)	99/159 (62.3)	0.039**
Restless legs *N = 500, *N* (%)	57/121 (47.1)	169/379 (44.6)	0.628	158/340 (46.5)	68/160 (42.5)	0.405
Dizziness *N = 497, *N* (%)	48/119 (40.3)	115/378 (30.4)	0.045**	109/336 (32.4)	54/161 (33.5)	0.807
Headache *N = 501, *N* (%)	68/121 (56.2)	193/380 (50.8)	0.300	177/339 (52.2)	84/162 (51.9)	0.940
Muscular pain *N = 497, *N* (%)	84/120 (70.0)	247/377 (65.5)	0.365	227/338 (67.2)	104/159 (65.4)	0.699

*N refers to the number of participants responding to the questions of symptoms.

**These p-values do not reach the level of significance when correcting for multiple hypothesis testing.

The length of the alimentary limb was 100 cm in 440 (83%) of the patients, and 65 (15%) women and 25 (23%) men with preoperative BMI >50 kg/m^2^ had an alimentary limb of 150 cm (*p* = 0.05). There were no differences in the distribution of ferritin levels according to limb length; however, 29 (32%) of those with long limbs had ferritin < 15 µg/L, compared to 96 (21.8%) in those with an alimentary limb of 100 cm (n.s.).

To evaluate whether ferritin levels were influenced by inflammation, C-reactive protein (CRP) levels were analyzed. As 88% of the participants had CRP ≤ 5 mg/L, and 96% had CRP ≤ 10 mg/L, further analyses on the relationship between ferritin levels and inflammation, including transferrin receptor levels in this population, were not performed.

When analyzing the relationship between testosterone levels and hemoglobin, the 26 men with anemia (Hgb <13.7 g/dl) had lower testosterone levels than the 81 men without anemia, at 14.0 (7.0) nmol/L vs. 17.4 (6.2) nmol/L (*p* < 0.05), but this did not amount to a significant difference when correcting for multiple hypothesis testing. Only one participant reported using testosterone supplement. There was no relationship between testosterone levels and hemoglobin in women.

## Discussion

The main findings in this study were that the frequencies of iron deficiency and iron insufficiency were common a decade or more after RYGB even when the patients were advised to use iron supplements either continuously or intermittently to keep the ferritin level above 50 µg/L. Intravenous iron treatment was available for patients with iron deficiency who did not respond to per oral treatment. However, this treatment was more often offered to women than men. The cost of intravenous iron treatment was covered by the public hospitals, but vitamin supplements and oral iron supplements were paid by the patients.

The frequency of anemia was just as common among men as among women when gender-specific cutoff levels were used. As previously reported by Monaco-Ferreira and Leandro-Merhi, the hemoglobin levels did not differ between patients using per oral iron supplements and patients who did not (35). The interquartile ranges of ferritin were also identical for the two groups. Whether this results from only patients with low iron stores being recommended supplements, or whether per oral iron supplements had little effect, remains to be explored. A recently published systematic review reveals a high variation in current strategies on iron supplementation following bariatric surgery; many of them do not prevent development of iron deficiency and iron deficiency anemia (27). In a non-bariatric population, per oral iron supplements once or twice a day have been reported to decrease iron absorption due to temporally increased levels of hepcidin, and iron supplements in lower doses or alternate days might be more effective (36). After RYGB, suggestions have been made to evaluate the potential response to oral iron supplements by using hepcidin concentration and transferrin saturation (37). The daily dose of iron supplements was not registered in the BAROBS study, and hepcidin concentration tests were not available at the laboratories at the participating hospitals.

Iron deficiency in the general population is common; ferritin below 15 µg/L was reported for 11.8% in a general health study from Germany with 5,000 adult participants (38). An even higher frequency of iron deficiency is found in bariatric surgery candidates (39). Obesity-related conditions such as non-alcoholic steatohepatitis (NASH) and low-grade inflammation might affect iron absorption and the frequency of iron deficiency and anemia prior to bariatric surgery (40). Obesity is also associated with hematologic changes other than iron deficiency and anemia (41).

According to a systematic review by Enani et al. including 1897 RYGB patients with a follow-up of 27.8 (range 12–120) months, the incidence of iron deficiency was 12.9% before RYGB surgery and 24.5% post-RYGB, and the incidence of anemia due to iron deficiency was 16.7% post-RYGB (42). The same level of anemia was reported in a French survey using administrative data (1). Although the follow-up period in the present study was longer, the frequency of anemia was at the same level in women, but it was found to be higher in men. However, compared to studies with more than 5 years follow-up after RYGB, the frequency of anemia was lower in the present study than reported by Kotkiewicz et al., who found a 58% risk of anemia 7 years after surgery (33).

Obinwanne et al. reported 53% of RYGB patients having ferritin <50 µg/L at some stage during the observation period of 10 years, and a mean hemoglobin level of 11.9 g/dl. The present study found that even more patients had ferritin <50 µg/L at some stage during follow-up, but the hemoglobin levels were higher. Socio-economic conditions and patient use of public versus private health service might play roles in the nutritional status long-term after bariatric surgery.

The analyses of diffuse self-reported symptoms that might be associated with iron deficiency found no substantial relationships. The questions regarding symptoms were likely too general to reveal an association, and several other factors in life after bariatric surgery might cause identical symptoms.

The clinical implication of iron deficiency without anemia in a post-bariatric population is poorly understood, and the relationship between iron deficiency and diffuse symptoms impairing self-experienced health and quality of life needs further investigation.

Long-term follow-up is necessary to detect iron deficiency and prevent anemia after bariatric surgery, and this is especially important for women who are planning a pregnancy after bariatric surgery, as prenatal iron deficiency can affect the fetus adversely (43). In addition, during long-term follow-up after bariatric surgery, other causes of iron deficiency and anemia must be kept in mind, the most common being heavy menstrual bleeding in premenopausal women, and gastrointestinal bleeding in men and women after menopause.

Further investigation is needed to explain why some patients have normal ferritin levels although they do not report taking specific iron supplements. The compliance to a recommended diet and general supplements after bariatric surgery fell outside the scope of this study.

## Strengths and Limitations

The strengths of this study were that the RYGB procedure was performed identically at the three participating hospitals and more than half of the patients who underwent RYGB in a given period met to a clinical follow-up more than 10 years after surgery.

The fact that the participation rate was less than 60% of eligible patients might be a limitation on the interpretation of the results of this study; however, there was no difference in sex and age among participants and the study population.

Further, the lack of a comparison group and self-reported information on iron supplement use were other limitations to the study, as well as the fact that information on symptoms that could be associated with iron deficiency had not been collected prospectively, neither had information on menstrual bleeding among premenopausal women.

This study was performed in a prosperous country with public health services covering all citizens. Although the socioeconomic status has been found to be lower among bariatric surgery candidates than the general population, most patients can afford the recommended postoperative supplements, and all patients receive reimbursement for intravenous iron treatment. In addition, the number of vegetarians is low in this population, implying that red meat is an important source of iron before as well as after bariatric surgery. Contraceptives that minimize menstrual bleeding were common among women before menopause in the study population. All of these facts may reduce the generalizability of the findings of the study.

## Conclusion

Ferritin below the level recommended, at 50 µg/L, occurred in two-thirds of the patients 10 years or more after RYGB, although more than half reported taking per oral iron supplements, and one out of four had received intravenous iron treatment. The frequency of anemia is just as high in men as in women, when gender-specific thresholds are applied, and women have a lower frequency of anemia than reported in other studies reporting long-term results after gastric bypass surgery.

## Data Availability Statement

The raw data supporting the conclusions of this article will be made available by the authors, without undue reservation.

## Ethics Statement

The studies involving human participants were reviewed and approved by the Regional Ethics Committee of Norway REK 2017/1828 REK south-east B. The patients/participants provided their written informed consent to participate in this study.

## Author Contributions

All authors contributed to conception and design of the study. JS and CK performed the statistical analysis. JS wrote the first draft of the manuscript. All authors contributed to the article and approved the submitted version.

## Funding

This work was supported by the Liaison Committee for education, research, and innovation in Central Norway and funding from all three local hospital trusts (Grant number 46055500).

## Conflict of Interest

The authors declare that the research was conducted in the absence of any commercial or financial relationships that could be construed as a potential conflict of interest.

## Publisher’s Note

All claims expressed in this article are solely those of the authors and do not necessarily represent those of their affiliated organizations, or those of the publisher, the editors and the reviewers. Any product that may be evaluated in this article, or claim that may be made by its manufacturer, is not guaranteed or endorsed by the publisher.
